# A Rare Case of Polymyositis and Systemic Sclerosis Overlap Syndrome: Diagnosis and Treatment

**DOI:** 10.7759/cureus.36434

**Published:** 2023-03-20

**Authors:** Mohammad K Uddin, Roopeessh Vempati, Sravani Bhavanam, Praver Chandan C Parven, Chinmay Khotele, Raja H Chitturi, Smaran Kasireddy, Mahak Bhandari, Sweta Sahu

**Affiliations:** 1 lnternal Medicine, Gandhi Medical College & Hospital, Hyderabad, IND; 2 Internal Medicine, Gandhi Medical College & Hospital, Hyderabad, IND; 3 Internal Medicine, Sri Devaraj Urs Medical College, Kolar, IND; 4 Internal Medicine, Indira Gandhi Government Medical College & Hospital, Nagpur, IND; 5 Internal Medicine, Great Eastern Medical School and Hospital, Visakhapatnam, IND; 6 Internal Medicine, Jagadguru Jayadeva Murugarajendra (JJM) Medical College, Davanagere, IND; 7 Medical Student, Lokmanya Tilak Municipal Medical College, Mumbai, IND

**Keywords:** systemic sclerosis, myositis-mg overlap, sle-myositis overlap syndrome, polymyositis, diffuse systemic sclerosis

## Abstract

Diffuse scleroderma is a kind of scleroderma in which the immune system malfunctions, leading to excessive production of collagen in the skin and a variety of organ abnormalities. Based on previously recognized criteria, overlap syndrome is a disorder in which two or more medical illnesses are documented in a single patient. These syndromes are significantly more prevalent in illnesses with mixed connective tissue. In this case report, we present a patient with overlapping systemic sclerosis and polymyositis symptoms. The treatment and diagnosis of this extremely uncommon condition are discussed in further detail.

## Introduction

Connective tissue diseases refer to a spectrum of medical conditions that affect the protein-rich tissue that supports various organs and body parts, such as fat, bone, and cartilage. While these disorders primarily impact joints, muscles, and skin, they can also affect other organs like the eyes, heart, lungs, kidneys, gastrointestinal tract, and blood vessels. About 1 in 20 people with rheumatic complaints had signs of more than one rheumatic disorder in addition to their main diagnosis. So, the idea of undifferentiated connective tissue syndrome (UCTS) or overlap syndrome was made to describe these kinds of patients in clinics [1]. It is important not to miss the diagnosis of overlap syndromes because the outlook and treatment are very different from those of connective tissue disorders that happen on their own.

Systemic sclerosis (SSc) is a disease of the connective tissues that causes too much collagen to build up in the skin and internal organs and causes the blood vessels to react too strongly and block small blood vessels. Patients with SSc are put into three groups based on how much their skin is affected: limited SSc (lSSc), in which the skin is not affected at all; limited cutaneous SSc (lcSSc), in which the skin is affected mostly on the hands and face; and diffuse SSc (dSSc), in which the skin is affected from head to toe [2]. SSc patients can have myopathies anywhere from 5% to 81% of the time, depending on how muscle involvement is defined. Myositis in people with SSc looks and acts like it does in people with polymyositis (PM). This is why it is called scleroderma/polymyositis overlap (SSc-PM). Studies have shown that there is an increased rate of mortality and myocardial muscle involvement in the SSc-PM overlap compared to SSc. Therefore, it seems clinically relevant to identify SSc-PM overlap for close monitoring and early treatment [3]. The heterogeneity of pathological muscle findings, which includes the stigma of microangiopathy as well as an inflammatory infiltrate in approximately half of the cases and interstitial fibrosis, suggests that the pathophysiological process that leads to SSc-associated myopathy is likely to be complex. This is due to the fact that SSc-associated myopathy is associated with a variety of pathological muscle findings. There have been a number of contradictory findings published in reference to the link between clinicopathological presentation and pathological muscle characteristics [1].

## Case presentation

A 40-year-old female with a history of hypertension and diabetes mellitus presented to the hospital with joint pain and changes in the color of her skin for one year. She also complained of fatigue for six months, weakness for four months, burning pain in the chest for three months, and shortness of breath for three weeks.

The patient did not seem to have any symptoms a year ago, but then she started to have pain and stiffness in her joints. The onset of joint pain was insidious and symmetric. It had a waxing and waning course affecting more than five joints in the upper and lower limbs associated with redness, swelling, and a low-grade fever which was relieved on medication. Stiffness in the hands was of insidious onset and gradually progressive to the extent that she had difficulty holding a glass, which did not relieve with activity; stiffness in the knees and elbows improved with activity. There was no history of petechiae, purpura, or rashes. There were no complaints or evidence of a change in color of the skin with exposure to different temperatures. There was no history of dryness in the eyes or mouth. She lost 20 kg weight in six months. She had a history of weakness for four months, which was insidious in onset and limited her ability to comb her hair; initially, she had difficulty climbing stairs; difficulty in standing up from a squatting position; eventually, she was not able to get up from a chair. She had no history of loss of consciousness, trauma, or seizures. She reported burning pain in the chest for three months that was insidious onset, intermittent type, non-radiating, aggravated on lying down, relieved on taking medications, and was associated with bloating and postprandial nausea. She had grade 4 New York Heart Association (NYHA) shortness of breath for three weeks. It started slowly and got worse over time. She also had orthopnea, paroxysmal nocturnal dyspnea, and palpitations. Pedal edema was present for two weeks, extending up to the knee and later progressing to the face. No history of hematuria was observed. Her personal history revealed a decreased appetite and inadequate sleep. On physical examination, the patient appeared conscious, coherent, and oriented to time, place, and person. The patient was moderately built and poorly nourished, with signs of pallor. Bilateral pitting pedal edema extending up to the knee and facial puffiness were observed.

In the emergency room, she was found to have a blood pressure of 140/70 mmHg in the right arm in the sitting position, her heart rate was 72 beats per minute, and respiratory rate was 25 breaths per minute with a thoracoabdominal pattern. She was afebrile and had an oxygen saturation of 90% on room air. Elevated jugular venous pressure and venous hum were recorded. General random blood sugar (GRBS) was 218 mg/dl.

**Figure 1:**
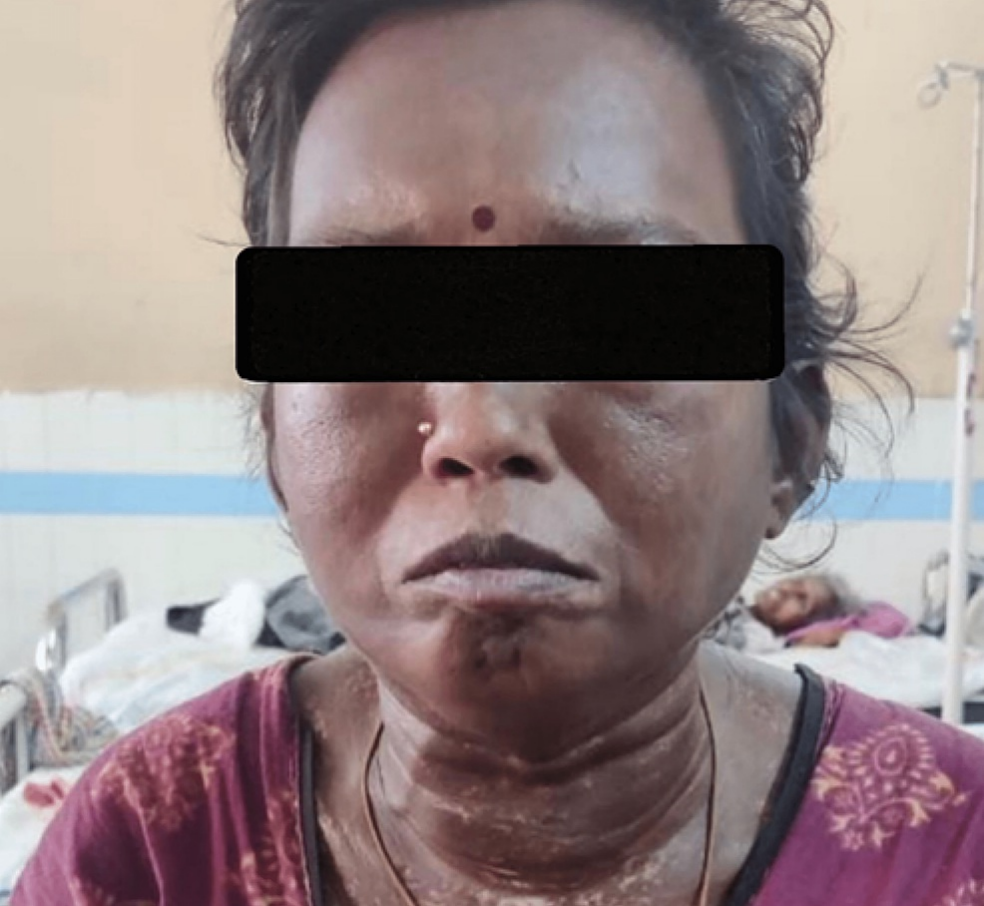
Thickening of neck region with salt and pepper pigmentation, low volume of hair

**Figure 2:**
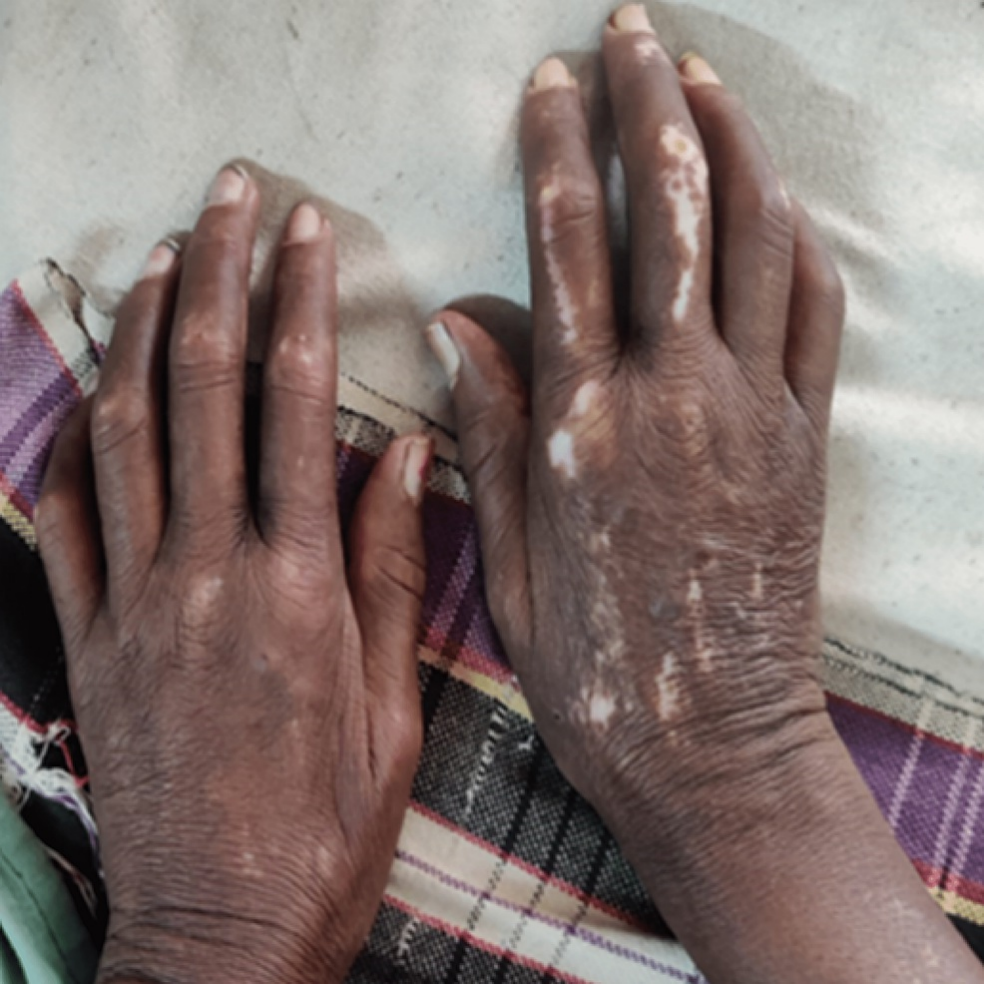
Salt and pepper pigmentation of the hands

Alopecia, microstomia (only incisors were visible on a wide opening of the mouth), the scleroderma neck sign, as well as salt and pepper pigmentation in the trunk and limbs, were noticed. Skin thickening (sclerodactyly) was seen on the face, neck, chest, upper abdomen, thighs, and the sides of the arms, hands, and fingers. There were no signs of calcinosis cutis, telangiectasia, ulcers, malar rash, or parotid enlargement (Figures 1, 2).

The apex beat could be felt at the left fifth intercostal space, 1 cm to the side of the midclavicular line. There were no heart sounds or thrills that could be felt. Chest expansion is reduced. On auscultation, a loud P2 and a high-pitched early diastolic murmur were best heard over the left sternal border, radiating to the apex. The intensity increased with inspiration and decreased with Valsalva in the pulmonary area, which pointed to pulmonary regurgitation. A pansystolic murmur was present in the tricuspid area, with intensity increasing with inspiration and decreasing with Valsalva, consistent with tricuspid regurgitation. Normal heart sounds were heard in the aortic and mitral areas, and an S3 sound was heard. Normal bilateral vesicular sounds are heard, with bilateral end-inspiratory crepitations heard over the interscapular and infraaxillary areas. Musculoskeletal examination revealed positive prayer sign, scissors, and squeeze tests. Muscle strength was decreased (3/5 in all four limbs), and deep reflexes were 1+, while superficial reflexes were normal.

**Figure 3:**
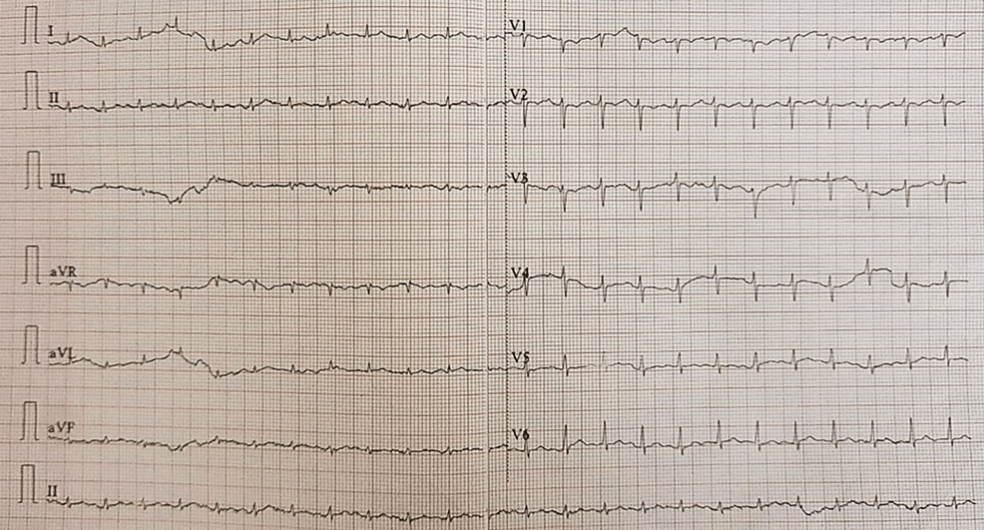
Sinus tachycardia

On investigation, a complete blood picture revealed anemia, specifically anisocytosis and microcytic hypochromic anemia, with a hemoglobin level of 3.9 g/dl. The iron profile showed that the level of ferritin was lower and the level of total iron-binding capacity (TIBC) was higher, implying that the patient had iron deficiency anemia. The direct Coombs test (DCT) and the indirect Coombs test (ICT) tests were negative. Urinalysis revealed albuminuria (2+). The liver function test (LFT) and renal function test (RFT) were within normal limits. The arterial blood gas (ABG) test showed a pH of 7.478 and pCO2 of 19.1 mm Hg. Ultrasound of the abdomen revealed coarse echotexture of the liver, grade 1 renal parenchymal changes, and mild pleural effusion and consolidation of the underlying lung. A two-dimensional echocardiography (2D ECHO) revealed a dilated right atrium and right ventricle with pulmonary and mild tricuspid regurgitation, a right ventricular systolic pressure (RVSP) of 32 mmHg, and severe pulmonary arterial hypertension (PAH). Pulmonary function tests revealed increased forced expiratory volume in the first second/forced vital capacity (FEV1/FVC) and normal diffusing capacity of the lungs for carbon monoxide (DLCO). The thyroid profile showed elevated thyroid stimulating hormone (TSH) with normal T3 and T4. Ultrasonography (USG) of the neck was consistent with chronic thyroiditis and Thyroid Imaging Reporting and Data System (TI-RADS)-1 lesion in bilateral lobes, likely a colloid cyst. An electrocardiogram (ECG) showed sinus tachycardia (Figure 3). Auto-immune serology tests indicated positive anti-centromere and anti-Jo-1 antibodies (Table 1).

**Table 1:** Auto-immune serology tests results RNP-sM - small nuclear ribonucleoprotein; Sm Antigen - Smith antigen; SSA - Sjögren's-syndrome-related antigen A; SSB - Sjögren's syndrome type B; PCNA - proliferating cell nuclear antigen; dsDNA - double-stranded deoxyribonucleic acid; AMA M2 - anti-mitochondrial M2

Antigen	Intensity	Class	Result
RNP-sM	4	0	Negative
Sm	0	0	Negative
SSA	1	0	Negative
Ro-S2 recombinant	3	0	Negative
SSB	2	0	Negative
SB	1	0	Negative
PM100	0	0	Negative
Jo-1	14	+	Positive
Centromere B	10	+	Positive
PCNA	3	0	Negative
dsDNA	2	0	Negative
Neurosomes	0	0	Negative
Histones	0	0	Negative
Ribosomal P protein	3	0	Negative
AMA M2	2	0	Negative
Co	2	0	Negative

**Figure 4:**
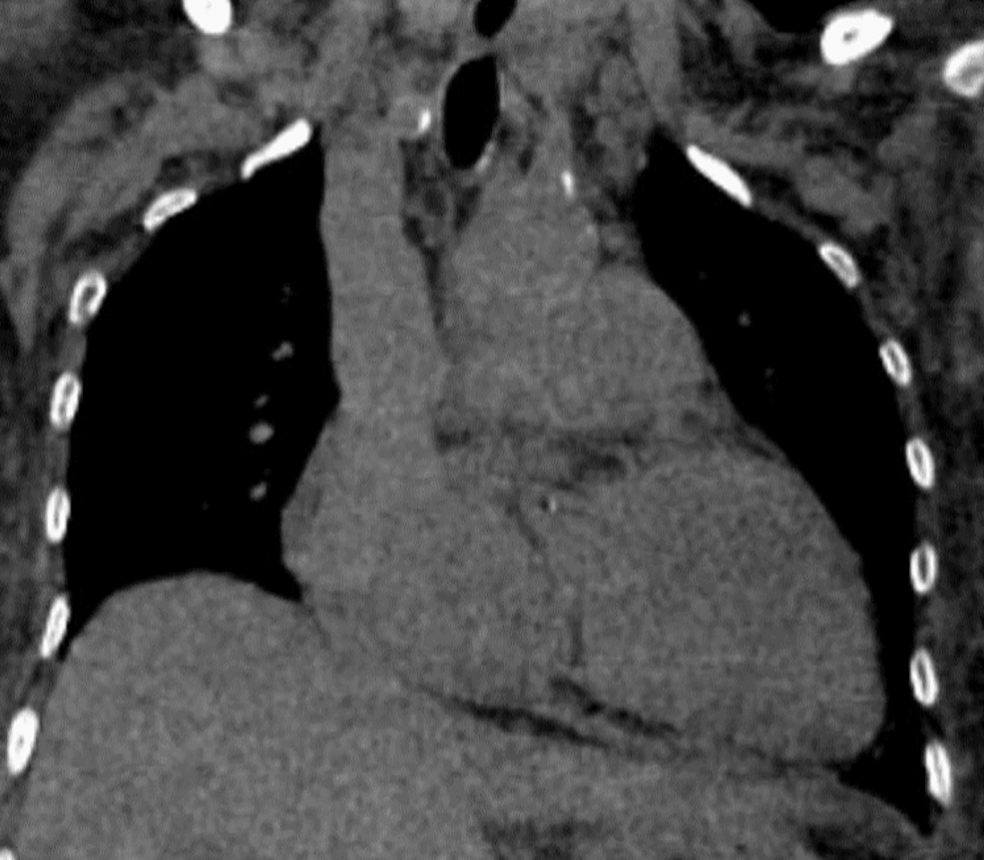
CT scan of the chest in the coronal plane demonstrating cardiomegaly

**Figure 5:**
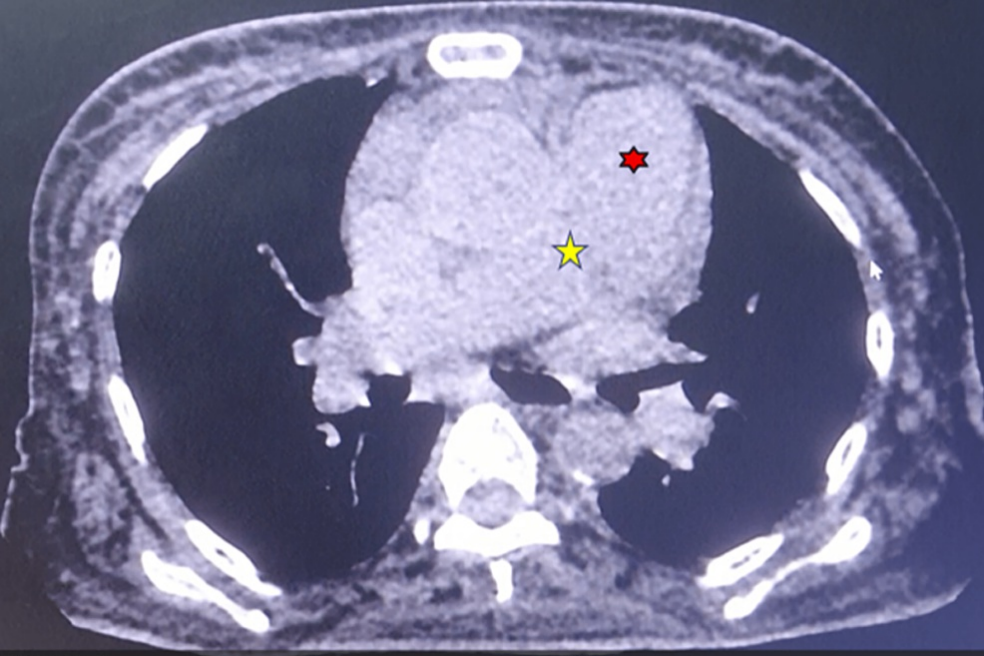
CT scan of the chest showing a dilated main pulmonary trunk (red) and right branch of the pulmonary artery (yellow)

**Figure 6:**
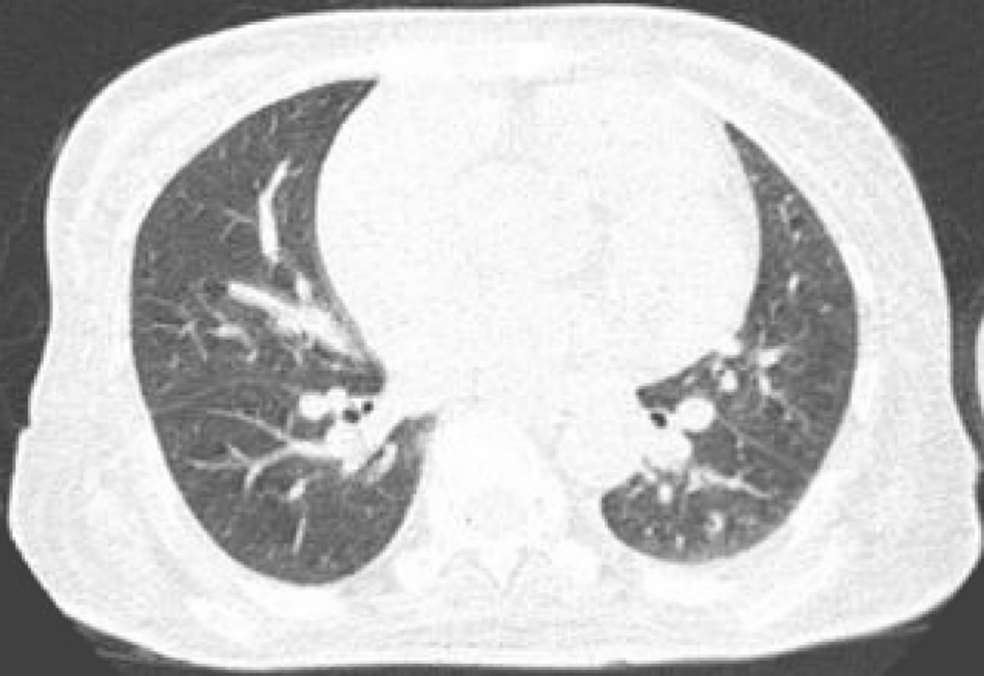
Cardiomegaly and early ILD changes ILD - interstitial lung disease

High-resolution computed tomography (HRCT) showed minor paraseptal emphysematous changes in the apical segment of the right upper lobe and the lateral basal segment of the right lower lobe. In addition to cardiomegaly (Figure 4), a dilated pulmonary trunk (32mm) and dilated right branch (26mm) (Figure 5) of the pulmonary artery were found, with a normal caliber of the left branch (22mm). HRCT also revealed early ILD changes (Figure 6). Chest X-rays revealed cardiomegaly, interstitial thickness, and perihilar opacities.

The patient was brought to the hospital for emergency care, where she was administered two units of packed red blood cells (PRBC), and an intravenous injection of furosemide (20 mg) twice daily (BD) was given for 10 days along with enalapril 5 mg per oral (PO), and BD was given throughout the stay. Injection-based biphasic insulin 20 U (12U/8U) was given for 10 days, along with metformin 500 mg BD, as an anti-diabetic regimen with regular general random blood sugar (GRBS) monitoring. Methylprednisolone injections of 1 gm IV OD were given for five days. After that, the dose was lowered to 80 mg IV OD (once daily) for five days, and then it was changed to 32 mg PO OD as the regular dose. Hydroxychloroquine 200 mg PO BD, mycophenolate mofetil (MM), cyclophosphamide, tadalafil 40 mg PO OD, ambrisentan 10 mg OD, and calcium vitamin D3 PO OD were also added to her regimen. By day 13, two more units of PRBC had been transfused, and the hemoglobin level had risen to 9.8 g/dl. Levothyroxine 50 mcg PO OD was prescribed after the de novo diagnosis of hypothyroidism. After 14 days in the hospital, the patient was discharged with methylprednisolone 32 mg tablets (OD), hydroxychloroquine 200 mg tablets (BD), enalapril 5 mg tablets (OD), tadalafil 20 mg tablets (OD), ambrisentan 40 mg tablets (OD), and advised to continue levothyroxine 50 mcg tablets (OD). After long-term follow-up, the patient died as she developed scleroderma renal crisis.

## Discussion

In this study, we describe a case of overlap syndrome. We believe the importance of the overlap syndrome is based on the relatively poor prognosis that these patients have in comparison to patients who just present with diffuse sclerosis [2]. Myositis changes under microscopy usually showed necrotic muscle fibers, lymphocytic infiltrates, and fibrosis, which are the most common findings under histopathology [3]. Overlap syndrome between diffuse scleroderma and polymyositis can be determined as a collection of signs existing between two pathologies that might exist either at the same time or followed by each other [4]. The core understanding of this pathology is still quite muddy, and no definitive answers have been found. Our case was diagnosed based on the European Alliance of Associations for Rheumatology (EULAR) and American College of Rheumatology (ACR) criteria of 2013, and antibody testing showed anti-Jo positivity, which demonstrated the possibility of polymyositis along with the features consistent with systemic sclerosis [5].

This poor prognosis seen in these types of patients could be due to pathological processes that involve fibrosis and vasculitis, as well as higher rates of degeneration of smooth muscle tissue in the lungs and other parts of the body. This has been said in other papers; cases of overlap syndrome also had a higher rate of heart involvement, pulmonary fibrosis, diffuse pigmentation of the skin, and contractures, which shows how important it is to find a way to diagnose it [6]. Some studies have shown that antinuclear ribonucleoprotein (anti-RNP) and anti-PM/SCL antibodies are better at diagnosing myositis. Anti-centromere antibodies, on the other hand, have been shown to protect people from getting myositis [7, 8]. But this shows how important it is to come up with more specific criteria for these conditions since myositis is often misdiagnosed when there are a lot of them. Even though these anticentromere antibodies are more often found in people with limited sclerosis, they have also been found in people with diffuse sclerosis, which has a more gradual start and spread and affects fewer organs. This is a bit of a puzzle since our case involved the whole body in a severe and widespread way and had antibodies. This made the case quite unique and different from what we know about anticentromere antibodies in systemic sclerosis [9, 10].

On closer inspection, we saw more changes in the lungs, such as bilateral pleural effusion and emphysema, on the high-resolution computed tomography (HRCT) scan. Most cases of pulmonary involvement fall into two groups: primary pulmonary disease, which can lead to pulmonary hypertension; and secondary pulmonary disease, which can be caused by a number of things, such as broncho-aspiration caused by gastroesophageal reflux, toxicity from medications, infections, and other things [11]. In our case, the signs of pulmonary hypertension can also be explained by the fact that we tested positive for anti-centromere B, which has been linked to a form of PAH that is less obvious and doesn't cause any symptoms [12]. Interstitial lung disease and anemia could have made her pulmonary hypertension worse, which led to her main complaint of being short of breath. In many cases of systemic sclerosis, anti-topoisomerase has been shown to be a key factor in determining the risk of ILD as well as the severity of the disease that the patient may eventually show [13]. In terms of treating the ILD itself, MMF and cyclophosphamide, as well as nintedanib (INBUILD trial), rituximab, and tocilizumab, have all been shown to be the new standards of treatment, but the availability and cost of these medications should be brought into the equation as well, as we try and determine the best course of action for our patient [14].

A further point of discussion would be the chronic anemia our patient faced throughout the treatment. The hemoglobin levels expressed in our case could possibly be due to a combination of factors, including a poor diet seen predominantly in women in the Indian subcontinent, along with other factors that could possibly be malabsorption from the gut due to fibrosis leading from diffuse scleroderma [15]. We are sure these could not be due to hemolytic anemia, even though this has been seen in certain case reports with similar presentations and antibody profiles [16]. Anemia is significant, as it has been proven to be a marker for more severe organ damage [17] in systemic sclerosis. Throughout the course of treatment, blood transfusions were provided, and while this provided some respite, it did not completely cure the patient, and in retrospect, this was probably the cause of worsening heart failure as well. New studies have shown that autologous stem cell transplant might be the way forward in these types of cases, as these have shown not only to improve the anemia but also to reduce skin manifestations in diffuse SSC, and these have been proven to be significantly better than cyclophosphamide [14].

Another interesting point could be the presence of thyroiditis in our patient, which has been shown to have a real clinical correlation [18]. Breg cells are a big part of the pathology of thyroiditis [19], but in our patient, there are also a number of other possible pathologies. There is no significant evidence of whether these rates are higher in overlap syndrome, and this could be another point of discussion in future studies and case reporting. Our patient was showing elevated TSH, which showed a lack of response to the doses of levothyroxine she was on, and further management could have been taken in this regard.

The outcome of this case shows how important it is to come to a common understanding and treatment plan for people with overlap syndrome. Due to the lack of knowledge about these conditions, patients tend to be misdiagnosed and end up being treated at later stages of their pathogenesis, when it is much harder to treat them because they affect so many different parts of the body, and the prognosis is usually worse. Our patient was given enalapril, which has been shown to increase the risk of scleroderma renal crisis [20]. Enalapril is one of the drugs that could help cover her pulmonary hypertension, but it has also been shown to increase the risk of a scleroderma renal crisis. Due to immunosuppression, methylprednisolone, mycophenolate mofetil (MM), and cyclophosphamide were given as first-line treatments for diffuse systemic sclerosis (SSc) [21].

## Conclusions

Overlap syndromes are common in rheumatic diseases, such as mixed connective tissue disorders. When a patient does not fit into a particular condition, a thorough evaluation should be performed, including autoimmune investigations, and then it should be determined whether the patient fits into any of the overlap syndromes documented in the medical literature. According to research and our case findings, these types of syndromes may be underdiagnosed, and as we have observed in our own case, the intensity of the symptoms and the rate of progression of the condition are rather frightening. Although antibodies are a valuable diagnostic tool, they are not perfect. Anticentromere antibodies were detected in our case of diffuse sclerosis, indicating that they are not yet complete. Because of this, we strongly recommend that individuals with systemic sclerosis-polymyositis (SSc-PM) have muscle biopsies, which can be utilized to determine whether SSc-PM overlap is occurring. In order to optimize treatment, increase survival, and improve the quality of life for these patients, as seen in our example where the patient presented with significant symptoms, we must ensure that we follow up with them frequently.
