# Correction: A Rare Case of Polymyositis and Systemic Sclerosis Overlap Syndrome: Diagnosis and Treatment

**DOI:** 10.7759/cureus.c108

**Published:** 2023-04-19

**Authors:** Mohammad K Uddin, Roopeessh Vempati, Sravani Bhavanam, Praver Chandan C Parven, Chinmay Khotele, Raja H Chitturi, Smaran Kasireddy, Mahak Bhandari, Sweta Sahu

**Affiliations:** 1 lnternal Medicine, Gandhi Medical College & Hospital, Hyderabad, IND; 2 Internal Medicine, Gandhi Medical College & Hospital, Hyderabad, IND; 3 Internal Medicine, Sri Devaraj Urs Medical College, Kolar, IND; 4 Internal Medicine, Indira Gandhi Government Medical College & Hospital, Nagpur, IND; 5 Internal Medicine, Great Eastern Medical School and Hospital, Visakhapatnam, IND; 6 Internal Medicine, Jagadguru Jayadeva Murugarajendra (JJM) Medical College, Davanagere, IND; 7 Medical Student, Lokmanya Tilak Municipal Medical College, Mumbai, IND

This article has been corrected at the request of the authors to revise the author list as follows: Anagha Shree has been removed from the author list and Mahak Bhandari has been added in the 8th author position. All authors have contacted the journal to request this change, which is the result of miscommunication among authors prior to submission. The submitting author greatly regrets the error and any inconvenience caused.

